# The role of academic mentorship and self-efficacy in enhancing self-directed learning readiness among undergraduate nursing students

**DOI:** 10.1186/s12912-026-04723-8

**Published:** 2026-05-14

**Authors:** Ghada Mohamed Attia Sherif, Samar AbdelRazek Younes ElSayed, Ahmed Abdelwahab Ibrahim El-Sayed, Ahmed Shaaban Attia, Naglaa Alsaied Mostafa Alfauomy

**Affiliations:** 1 Nursing Education Department, Faculty of Nursing, Damanhour University, Damanhour, Egypt; 2 Critical Care Nursing Department, Faculty of Nursing, Damanhour University, Damanhour, Egypt; 3 Nursing Administration Department, Faculty of Nursing, Alexandria University, Alexandria, Egypt; 4 Faculty of Nursing, Capital University, Cairo, Egypt; 5 College of Applied Medical Sciences, King Faisal University, Alhasa, Saudi Arabia; 6 Gerontological Nursing Department, Faculty of Nursing, Damanhour University, Damanhour, Egypt

**Keywords:** Academic mentorship, Self-efficacy, Self-directed learning readiness, Nursing students

## Abstract

**Aim/objective:**

This study aimed to examine the role of perceived academic mentorship and self-efficacy in enhancing self-directed learning readiness (SDLR) among undergraduate nursing students.

**Background:**

Self-directed learning readiness is essential for nursing students to effectively manage their learning in complex and evolving healthcare environments. While academic mentorship and self-efficacy have been associated with learning outcomes, their combined relationships with SDLR remain insufficiently explored.

**Methods:**

A descriptive correlational cross-sectional design was used. Data were collected from 500 undergraduate nursing students at the Faculty of Nursing, Damanhour University, Egypt, during the 2025–2026 academic year. Data were collected using three validated self-report scales measuring perceived academic mentorship, self-directed learning readiness, and self-efficacy. Data were analyzed using descriptive statistics, correlation, regression, and mediation analysis.

**Results:**

Perceived academic mentorship showed moderate positive correlations with self-directed learning readiness (*r* = 0.48, *p* < 0.01) and self-efficacy (*r* = 0.52, *p* < 0.01), while self-efficacy was also positively correlated with SDLR (*r* = 0.46, *p* < 0.01). Regression analysis indicated that demographic and academic variables explained 26.9% of the variance in SDLR. Mediation analysis revealed that self-efficacy partially mediated the relationship between perceived academic mentorship and SDLR (β = 0.15, 95% CI [0.10–0.20]).

**Conclusions:**

The findings indicate that students’ readiness for self-directed learning is not shaped by academic mentorship alone, but by how mentorship experiences translate into stronger beliefs in their ability to manage learning demands. This suggests that efforts to enhance students’ readiness for self-directed learning should prioritize mentorship approaches that move beyond guidance alone toward intentionally fostering students’ confidence, independence, and engagement in their learning processes.

**Clinical trial number:**

Not applicable.

## Introduction

Contemporary nursing practice requires graduates who can continuously update their knowledge, think critically, and function autonomously in complex clinical environments [1]. Accordingly, self-directed learning readiness (SDLR) has become a central outcome in nursing education, reflecting students’ preparedness to take responsibility for identifying learning needs, setting goals, and evaluating outcomes [2, 3]. Despite its importance, evidence indicated that nursing students demonstrate variable levels of SDLR, particularly in contexts transitioning from teacher-centered to learner-centered education [4, 5].

Among the determinants of SDLR, both academic mentorship and self-efficacy are consistently highlighted. Academic mentorship—embedded within formal university advisory systems—offers structured guidance, feedback, and developmental support, while self-efficacy influences students’ motivation, persistence, and engagement in learning [6–8]. Although each variable has been widely studied, research has rarely examined their combined and interrelated effects, limiting understanding of how educational structures and psychological mechanisms jointly shape SDLR.

This gap is particularly relevant in the Egyptian nursing education context, where formal academic mentorship systems are routinely implemented [9, 10], yet their role in fostering higher-order learning outcomes remains unclear. Specifically, it is not well established whether academic mentorship directly enhances SDLR or operates through mechanisms such as self-efficacy. Therefore, this study aims to examine the role of academic mentorship and self-efficacy in enhancing SDLR among nursing students, with attention to their interrelationships within a structured educational setting.

### Background and conceptualization of the study

Self-directed learning readiness is widely recognized as a foundational competency in nursing education, encompassing learners’ ability to take initiative, manage learning activities, and evaluate outcomes [11, 12]. Empirical studies have consistently demonstrated that higher SDLR is associated with improved academic performance, stronger critical thinking, and enhanced clinical competence [13, 14]. However, systematic reviews indicate that SDLR among nursing students is influenced by a combination of individual and contextual factors, rather than being an inherent or stable characteristic [15]. This highlights the need to examine both psychological and educational determinants of SDLR.

One of the most influential psychological determinants of SDLR is self-efficacy [16]. Rooted in social cognitive theory, self-efficacy shapes how students approach learning tasks, regulate effort, and respond to challenges [17, 18]. Students with high self-efficacy are more likely to initiate learning activities, persist in the face of difficulty, and utilize effective learning strategies—behaviors that directly underpin self-directed learning [19, 20]. In contrast, low self-efficacy may limit students’ engagement and reduce their ability to benefit from learning opportunities. Within nursing education, where students frequently encounter complex and demanding clinical and academic tasks, self-efficacy becomes a critical enabler of learning autonomy [21, 22]. Accordingly, self-efficacy can be conceptualized not only as a predictor of SDLR but also as a potential mechanism through which educational experiences influence learning outcomes.

While self-efficacy represents an internal mechanism, academic mentorship constitutes a key external or structural influence within the educational environment capable of shaping both self-efficacy and SDLR [23, 24]. In nursing education, mentorship has been shown to support students’ academic performance, facilitate professional socialization, and enhance learning engagement [25, 26]. Importantly, mentorship is not a uniform construct; its effectiveness depends on its structure and implementation [27].

In the context of Egyptian nursing education, academic mentorship is operationalized through a formal faculty-based advisory system, in which each student is assigned to a faculty member (e.g., assistant professor, associate professor, or professor) who assumes responsibility for academic guidance and student development [9, 10]. This role is distinct from that of a clinical instructor; while clinical instructors focus primarily on skill supervision in practice settings, academic mentors provide continuous academic advising, progress monitoring, feedback, and professional guidance across the academic program [28]. Mentors are typically assigned administratively based on departmental distribution rather than individual student characteristics, with each mentor overseeing a group of students and establishing an ongoing mentor–mentee relationship throughout the academic year [29]. Although the mentor–student ratio is not strictly standardized, mentors generally support multiple students within this structured advisory framework, ensuring continuous academic interaction and guidance [30].

Empirical evidence indicates that academic mentorship contributes to SDLR through multiple pathways. Studies have shown that mentorship provides direct instructional support by guiding students in planning, organizing, and evaluating their learning activities, which are essential components of self-directed learning [31, 32]. In addition, mentorship has been associated with the creation of supportive learning environments that promote reflection, autonomy, and progressive independence among nursing students [24, 34]. Importantly, several studies suggest that mentorship also strengthens students’ self-efficacy through mechanisms such as constructive feedback, encouragement, and role modeling, thereby enhancing their confidence in managing academic and clinical challenges [35–37]. Collectively, these findings indicate that academic mentorship may influence SDLR both directly and indirectly through its effect on self-efficacy, supporting the proposition that self-efficacy functions as a mediating mechanism in this relationship.

Despite these insights, several gaps remain in the literature. First, although SDLR, self-efficacy, and mentorship have each been studied extensively, few studies have examined them within an integrated framework, particularly in undergraduate nursing populations [38]. Second, existing research often reports associations without clearly explaining the mechanisms linking mentorship to SDLR, leaving uncertainty as to whether mentorship exerts its influence directly, indirectly, or through combined pathways [39, 40]. Third, there is limited empirical evidence from middle-income and Arab educational contexts, where structured academic mentorship systems are widely implemented but underexplored in relation to higher-order learning outcomes.

Taken together, these gaps indicate the need for a more comprehensive conceptual approach. In this study, SDLR is positioned as the primary educational outcome, influenced by academic mentorship as a structural factor and self-efficacy as a psychological mechanism. By examining both direct and indirect relationships among these variables within the context of Egyptian nursing education, the study seeks to provide a more nuanced understanding of how academic mentorship can be leveraged to develop self-directed, confident learners.

### Aim of the study

The study aimed to examine the role of perceived academic mentorship and self-efficacy in enhancing self-directed learning readiness among nursing students. Specifically, it seeks to determine whether higher levels of perceived academic mentorship and self-efficacy are associated with improved readiness for self-directed learning.

### Research hypotheses

#### H1

Nursing students who report higher levels of perceived academic mentorship will demonstrate significantly higher levels of self-directed learning readiness.

#### H2

Nursing students with higher levels of self-efficacy will demonstrate greater readiness for self-directed learning.

#### H3

Self-efficacy will mediate the relationship between perceived academic mentorship and self-directed learning readiness among nursing students.

## Methods

### Research design and setting

A descriptive correlational cross-sectional design was employed. The study was conducted at the Faculty of Nursing, Damanhur University in El-Beheira Governorate, Egypt, during the academic year 2024–2025, second term. It included nine academic departments that span various nursing fields: Adult Health Nursing, Critical Care and Emergency Nursing, Pediatric Nursing, Obstetric and Gynecological Nursing, Nursing Administration, Community Health Nursing, Gerontological Nursing, Nursing Education, and Psychiatric Nursing and Mental Health. Data were collected from undergraduate nursing students enrolled in the different academic levels.

### Sample size and study population

The study targeted undergraduate nursing students enrolled during the 2025–2026 academic year. A proportionate sampling approach was used to ensure representation of students across all academic years. Eligible participants were those who were currently enrolled in the program, willing to participate, assigned an academic mentor as part of the faculty’s academic advising system, and available at the time of data collection. Students in internship or graduate programs, as well as those unwilling to participate, were excluded from the study.

Sample size was determined using a power analysis (95% confidence level, expected frequency of 50% and a 5% margin of error), resulting in approximately 352 students. However, a total of 500 students were included to enhance the reliability, statistical power, and generalizability of the study findings, see Table 1.

**Table 1:** Distribution of study participants

Academic level	Total number of students	Number of students included in the study
First level	908	100
Second level	1129	136
Third level	933	113
Fourth level	1175	141
**Total**	**4145**	**500**

### Data collection tools

Four validated self-administered questionnaires were used:

#### Tool I: Demographic and academic characteristics

This tool was developed by the researcher based on a review of relevant literature and was designed to capture essential demographic and academic variables that may influence students’ learning experiences. It included items assessing age, sex, academic year, cumulative grade point average (CGPA), previous execution of academic assignments, and the extent to which students use technology in their studies. The item “previous execution of academic assignments” was measured as a dichotomous variable (Yes/No), indicating whether students had prior experience completing academic tasks. The item “number of academic assignments completed” was measured as a continuous variable, where participants reported the exact number of assignments they had completed during their academic studies. The “extent of technology use in study” was operationalized using a 4-point ordinal scale (rarely, sometimes, often, always), reflecting the frequency of using technology for academic purposes such as research and accessing electronic resources. These items were used to obtain a comprehensive overview of participants’ academic engagement and study-related behaviors.

#### Tool II: Advisory working alliance inventory—advisor version (AWAI–A)

The Advisory Working Alliance Inventory—Advisor Version was used to assess the quality of the advisory working alliance from the advisor’s perspective. The instrument was developed by Schlosser and Gelso)2005) [33] to evaluate the effectiveness of the advisor–student relationship in academic settings. The AWAI–A consists of 30 items that measure key dimensions of the advisory relationship across three domains: rapport (the emotional bond and mutual trust), apprenticeship (guidance and professional development), and identification–individuation (support for student autonomy and independence). Each item is rated on **a** 5-point Likert scale ranging from 1 (strongly disagree) to 5 (strongly agree). Higher scores indicate a stronger and more effective advisory working alliance. The reliability of the Arabic version of the instrument was assessed using Cronbach’s alpha coefficient, which indicated good internal consistency (α = 0.86).

#### Tool III: Self-directed learning instrument (SDLI)

The SDLI, developed by Cheng et al. (2010) [41], was used to assess students’ ability and readiness for self-directed learning. It is a 20-item self-report questionnaire, with responses rated on a 5-point Likert scale ranging from 1 (strongly disagree) to 5 (strongly agree). The instrument comprises four subscales: Learning Motivation (6 items), which evaluates students’ willingness and positive attitude toward learning; Planning and Implementing (6 items), which assesses the ability to set goals, design, and carry out learning plans; Self-Monitoring (4 items), which measures the ability to reflect on and adjust one’s learning process; and Interpersonal Communication (4 items), which captures the ability to seek feedback, collaborate, and share ideas with others. Total scores range from 20 to 100, with higher scores reflecting greater self-directed learning ability. The SDLI has demonstrated strong psychometric properties, including high internal consistency (Cronbach’s α = 0.92 overall; subscales ranging from 0.79 to 0.87) and good construct validity supported by factor analysis. It has been widely applied in nursing education research to evaluate baseline self-directed learning ability and the effectiveness of educational interventions. The reliability of the Arabic version of the instrument was assessed using Cronbach’s alpha coefficient, which indicated good internal consistency (α = 0.79).

#### Tool IV: General Self-Efficacy Scale (GSES)

Self-efficacy was measured using the General Self-Efficacy Scale (GSES) developed by Schwarzer and Jerusalem (1995) [42]. The scale consists of 10 items, each rated on a 4-point Likert scale (1 = not at all true to 4 = exactly true). It assesses individuals’ beliefs in their ability to cope with a variety of stressful situations and challenges. The total score ranges from 10 to 40, with higher scores reflecting greater self-efficacy. The GSES has been validated in multiple populations and languages, consistently showing high reliability (Cronbach’s α coefficients ranging from 0.76 to 0.90) and good construct validity. The reliability of the Arabic version of the instrument was assessed using Cronbach’s alpha coefficient, which indicated good internal consistency (α = 0.91).

### Study procedures

#### Tool preparation & pilot study

The research instruments were translated into Arabic by two independent bilingual translators who are proficient in both English and Arabic. The translators worked separately to ensure independent forward translations. A reconciliation process was then conducted by the research team to compare and merge the translated versions into a single harmonized Arabic version, resolving any discrepancies. To ensure linguistic equivalence, the reconciled Arabic version was back-translated into English by an independent translator who was blinded to the original English version. Discrepancies between the original and back-translated versions were reviewed and resolved accordingly.

The Arabic version was then evaluated by an independent panel of experts to assess content validity, clarity, and cultural appropriateness. Additionally, cognitive interviews were conducted with a purposive sample of 15 participants to evaluate item clarity, comprehensibility, and relevance. Feedback from these participants was used to refine the wording of the instrument. Following the validation process, reliability was assessed using Cronbach’s alpha to determine internal consistency. A pilot study involving 50 students was conducted to assess the clarity, readability, and feasibility of the instruments, as well as the time required for completion. The pilot participants were recruited using a convenience sampling technique from the same setting as the main study and were similar to the target population in terms of key demographic and academic characteristics. The results indicated that the questionnaire was clear and easy to understand, and it required approximately 10–15 min to complete. Data from the pilot study were not included in the final analysis.

### Data collection procedure

Approval for conducting the study was obtained from the Ethical Committee of the Faculty of Nursing, Damanhour University, in addition to formal permissions from the faculty administration and the heads of the nine academic departments. Data collection was conducted online between October and December 2025 in the academic year 2025–2026. A proportionate sampling approach was used to ensure representation of students across the four academic levels. Based on the enrollment distribution in each level, a predetermined number of students from each academic year were invited to participate to achieve a total sample of 500 students. Although the survey was distributed online and accessible to all students, participant recruitment was monitored continuously. The number of responses from each academic level was tracked, and data collection was closed for each level once the predetermined sample size was reached to ensure proportionate representation.

Before data collection, the researcher delivered an online orientation session explaining the study objectives, procedures, and the voluntary nature of participation. Participants’ questions were addressed, and confidentiality and anonymity were emphasized. The survey was administered electronically through Google Forms. Informed consent was obtained digitally, whereby participants had to select “Agree” to proceed; those who selected “Disagree” were automatically excluded. The survey remained open until the required number of participants from each academic level was reached, and periodic reminders were issued to minimize non-response.

### Ethical considerations

Approval for the study was obtained from the Research Ethics Committee of the Faculty of Nursing at Damanhour University, Egypt, in September 2025 (Reference No. 116-k). The study was conducted in accordance with the ethical principles of the Helsinki Declaration. Prior to participation, all individuals received comprehensive information regarding the study’s objectives and procedures, and written informed consent was secured from each participant. The research adhered to the principles of voluntary participation, anonymity, and confidentiality throughout the study. Participants were also informed of their right to withdraw at any time without any consequences.

### Data analysis

Data were coded, entered, and analyzed using the Statistical Package for Social Sciences (SPSS) version 26 (IBM Corp., Armonk, NY, USA). Prior to analysis, data were screened for completeness, accuracy, and normality. Descriptive statistics were used to summarize students’ sociodemographic and academic characteristics, as well as scale scores, including frequencies and percentages for categorical variables and means with standard deviations for continuous variables.

The internal consistency reliability of the study instruments was assessed using Cronbach’s alpha coefficients. Scale scores for mentorship effectiveness, self-directed learning readiness, and self-efficacy were calculated according to developers’ scoring guidelines, with higher scores indicating higher levels of the respective constructs. Inferential statistics were applied to test study hypotheses. Independent samples *t*-tests and one-way analysis of variance (ANOVA) were used to examine differences in mentorship, self-efficacy, and self-directed learning scores across demographic and academic groups. Post hoc comparisons were conducted when appropriate. Pearson’s correlation coefficient was employed to assess the relationships among mentorship quality, self-efficacy, and self-directed learning readiness.

Multiple linear regression analysis was performed to identify significant predictors of self-directed learning readiness while controlling for sociodemographic and academic variables. Model assumptions were checked prior to analysis. To examine the hypothesized mediating role of self-efficacy in the relationship between mentorship quality and self-directed learning readiness, mediation analysis was conducted using the regression-based approach. Direct, indirect, and total effects were estimated, and the significance of the indirect effect was evaluated using bias-corrected bootstrap confidence intervals (95%). Statistical significance was set at *p* ≤ 0.05.

## Results

Table 2 shows that the majority of students were female (65%), and most were aged 21–23 years. Female students reported higher mean scores in perceived academic mentorship, self-directed learning readiness, and self-efficacy compared to males, with statistically significant differences (t-test, *p* < 0.05). Scores differed significantly across age groups, with older students demonstrating higher mean scores across all three measures (one-way ANOVA, *p* < 0.05). Similarly, scores increased significantly across academic years, with senior students demonstrating higher levels across all three measures (one-way ANOVA, *p* < 0.01). Students with higher CGPA (≥ 3.5) showed significantly higher scores compared to those with lower CGPA (t-test, *p* < 0.05). In addition, greater use of technology for study was associated with higher scores in perceived academic mentorship, self-directed learning readiness, and self-efficacy, with statistically significant differences observed between groups (ANOVA, *p* < 0.001).

Table 3 demonstrates significant positive correlations among perceived academic mentorship, self-directed learning readiness, and self-efficacy. Academic mentorship showed moderate positive correlations with both self-directed learning readiness (*r* = 0.48, *p* < 0.01) and self-efficacy (*r* = 0.52, *p* < 0.01), indicating that stronger advisor–advisee working relationships are associated with higher levels of autonomous learning and confidence in personal abilities. Similarly, self-directed learning readiness was moderately correlated with self-efficacy (*r* = 0.46, *p* < 0.01), suggesting that students who are more self-directed tend to feel more capable and empowered in their academic performance. Overall, these results highlight a coherent pattern in which supportive advisory relationships contribute to enhanced learning autonomy and self-belief, reinforcing the interdependence between academic guidance, learning behaviors, and psychological resources.

Table 4 showed that sociodemographic and academic characteristics significantly predicted students’ self-directed learning scores. The model explained 26.9% of the variance in self-directed learning readiness (Adjusted R² = 0.269), indicating a moderate overall effect of the included predictors. Gender emerged as a significant contributor, with female students demonstrating higher levels of self-directed learning than males. This finding aligns with earlier research indicating that female nursing students tend to adopt more active and independent learning approaches. Academic year was also a significant predictor across the model, showing a steady progression in self-directed learning readiness levels from Year 1 to Year 4.

This suggests that as students advance in their academic program, they gain more confidence, autonomy, and experience in planning and managing their learning. The developmental nature of the curriculum and increasing exposure to clinical and professional environments may help explain this progression. CGPA exerted a strong positive influence on self-directed learning readiness, reflecting that students with higher academic achievement tend to be more organized, motivated, and independent in their learning behaviors. This finding reinforces the strong link between academic performance and the development of effective self-directed learning skills.

Table 5 reveals that mediation analysis suggests that the advisory working alliance (mentorship quality) exerts both a direct and an indirect effect on students’ self-directed learning, with general self-efficacy acting as a significant partial mediator. Specifically, stronger mentorship is associated with greater self-efficacy (a = 0.52, *p* < 0.001), and higher self-efficacy predicts greater self-directed learning when controlling for mentorship (b = 0.29, *p* < 0.001). The indirect effect (a × b = 0.15, 95% CI [0.10, 0.20], z = 5.63, *p* < 0.001) indicates that approximately 31.2% of the total association between mentorship and self-directed learning is transmitted via improvements in self-efficacy. The remaining direct effect of mentorship on self-directed learning was also statistically significant (c′ = 0.33, *p* < 0.001), which implies partial mediation—mentorship improves self-directed learning both by boosting students’ self-efficacy and through other direct pathways (e.g., modeling, scaffolding, practical guidance).

**Table 2:** Sociodemographic and academic characteristics of nursing students (*n* = 500)

Sociodemographic Variables	*n*	%	AWAI–A Total Mean ± SD	Test statistics *P*	SDLI Total Mean ± SD	Test statistics *P*	GSES Total Mean ± SD	Test statistics, *P*
**Age**								
18–20	150	30%	122.0 ± 15.8	**F = 6.72**,*p* = 0.001*	82.5 ± 9.8	**F = 5.98**,*p* = 0.003*	30.3 ± 4.6	**F = 4.12**,*p* = 0.017*
21–23	275	55%	125.8 ± 15.1		84.8 ± 10.0		31.5 ± 4.6	
24–28	75	15%	127.5 ± 14.9		86.0 ± 9.7		2.2 ± 4.5	
**Gender**								
Male	175	35%	121.5 ± 15.8	t = 2.15, 0.032*	81.7 ± 9.7	t = 2.05, 0.041*	30.2 ± 4.5	t = 2.05, 0.048*
Female	325	65%	126.5 ± 15.2		85.7 ± 10.0		31.9 ± 4.7	
**Academic Year**								
Year 1	125	25%	122.2 ± 16.3	F = 8.45, < 0.001*	82.1 ± 9.9	F = 7.92, < 0.001*	30.1 ± 4.8	F = 5.21, 0.002*
Year 2	125	25%	124.0 ± 15.6		83.5 ± 10.2		31.0 ± 4.7	
Year 3	125	25%	126.5 ± 14.8		85.0 ± 10.1		32.0 ± 4.5	
Year 4	125	25%	127.8 ± 14.7		86.2 ± 9.4		32.1 ± 4.6	
**CGPA**								
< 3.5	325	65%	121.2 ± 14.0	t = 2.98, 0.003*	81.4 ± 9.5	t = 2.72, 0.007*	30.6 ± 4.3	t = 2.51, 0.012*
≥ 3.5	175	35%	128.2 ± 16.0		86.2 ± 10.0		32.2 ± 4.6	
**Use of Technology in Study**								
High	300	60%	127.0 ± 15.2	F = 12.60, < 0.001*	85.9 ± 10.1	F = 11.85, < 0.001*	32.0 ± 4.5	F = 10.90, < 0.001*
Moderate	150	30%	123.0 ± 15.5		83.5 ± 9.8		31.0 ± 4.7	
Low	50	10%	118.5 ± 14.9		80.0 ± 9.6		29.5 ± 4.2	

F: Anova test, t: t-test, *SD: Standard Deviation; *p* ≤ 0.05; AWAI–A: Advisory Working Alliance Inventory—Advisor Version; SDLI: Self-Directed Learning Instrument; GSES: General Self-Efficacy Scale

**Table 3:** Pearson correlation between study variables (*n* = 500)

Variables	1	2	3
1. Academic mentorship		*r* = 0.48 (*p* < 0.01*)	*r* = 0.52 (*p* < 0.01*)
2. Self-Directed Learning reediness			*r* = 0.46 (*p* < 0.01*)
3. Self-Efficacy			

*p < 0.01

**Table 4:** Multiple linear regression analysis predicting self-directed learning readiness among nursing students (*N* = 500)

Variables	Β	SE	T	*P*
**Constant (Intercept)**	75.200	3.120	24.103	< 0.001*
Gender (Male vs. Female)	3.325	1.012	2.784	0.006*
Academic Year (Year 1 vs. Year 2)	1.402	0.522	2.685	0.008*
Academic Year (Year 2 vs. Year 3)	1.512	0.516	2.931	0.004*
Academic Year (Year 3 vs. Year 4)	1.279	0.498	2.567	0.011*
CGPA (< 3.5 vs. ≥3.5)	4.810	1.108	4.342	< 0.001*
Use of Technology (Low vs. Moderate)	3.554	0.884	4.018	< 0.001*
Use of Technology (Moderate vs. High)	2.461	0.733	3.354	< 0.001*
**R** ^**2**^	0.281			
**Adjusted R** ^**2**^	0.269			

Dependent variable: Self-Directed Learning Instrument (SDLI) total score. β: Unstandardized regression coefficient; SE: Standard error. *p ≤ 0.05 indicates statistical significance

**Table 5:** Mediation analysis — effect of self-efficacy between perceived academic mentorship and self-directed learning readiness (*n* = 500)

Path	B (standardized)	95% CI	t	*p*
**Direct effects**				
Academic mentorship → Self-Efficacy (a)	**0.520**	(0.445–0.595)	13.59	< 0.001*
Self-Efficacy → Self-Directed Learning readiness (b, controlling for X)	**0.288**	(0.197–0.380)	6.19	< 0.001*
Academic mentorship → Self-Directed Learning readiness (total effect, c)	**0.480**	(0.403–0.557)	—	< 0.001*
**Indirect effect**				
Academic mentorship → Self-Efficacy → Self-Directed Learning readiness (a × b)	**0.150**	(0.098–0.202)	z = 5.63	< 0.001*
**Direct (partial) effect**	Academic mentorship → Self-Directed Learning readiness (c′)	**0.330**	(0.237–0.423)	—
**Total effect**	Academic mentorship → Self-Directed Learning readiness	**0.480**	(0.403–0.557)	—
**Proportion mediated**	(indirect / total)	**31.2%**	—	—

B values represent standardized regression coefficients. The mediation analysis was conducted using bootstrap resampling (5,000 samples) to estimate 95% confidence intervals. The model controlled for gender, academic year, CGPA, and use of technology. *p ≤ 0.05 indicates statistical significance

## Discussion

This study examined the interrelationships among perceived academic mentorship, self-efficacy, and SDLR among undergraduate nursing students. The findings provide a more integrated understanding of how relational and psychological factors are associated with students’ readiness for self-directed learning, moving beyond isolated associations to explore potential underlying mechanisms.

The findings indicate that perceived academic mentorship is positively associated with SDLR. This suggests that students who perceive more supportive and effective mentor–mentee relationships tend to report greater readiness to engage in self-directed learning. Rather than implying a direct causal influence, this relationship may reflect the role of academic mentorship as a contextual and relational resource that supports students’ engagement with learning processes. Through ongoing academic guidance, feedback, and interaction, mentorship may help students orient themselves within the learning environment and develop approaches to managing their academic responsibilities. Similar associations have been reported in prior studies linking mentorship with improved academic engagement, adjustment, and learning behaviors in nursing education [9, 23].

However, the interpretation of this relationship requires careful consideration. Evidence from systematic reviews suggests that mentorship effectiveness varies considerably depending on mentor competence, consistency, and the nature of interaction [25, 39]. In some contexts, mentorship may inadvertently reinforce dependency if it is overly directive or lacks opportunities for student autonomy. The present findings contribute to this discussion by indicating that it is not merely the existence of mentorship, but how it is perceived and experienced by students, that is associated with SDLR. This shifts the emphasis from structural provision to relational and experiential dimensions of mentorship, extending existing literature by highlighting the importance of students’ subjective engagement with mentorship processes.

The study also found that self-efficacy is positively associated with SDLR, reinforcing its role as a key psychological determinant of learning behavior. Students with higher self-efficacy demonstrated greater readiness for self-directed learning, which may reflect their confidence in initiating, managing, and sustaining learning activities. This interpretation is consistent with social cognitive theory and supported by empirical studies demonstrating that self-efficacy influences goal setting, persistence, and self-regulation [17, 18]. In the context of nursing education, where students are required to navigate complex academic and clinical demands, self-efficacy may function as a critical enabler of adaptive learning behaviors [43].

At the same time, alternative perspectives emphasize that self-directed learning is shaped not only by individual beliefs but also by institutional and curricular structures [10, 44]. From this viewpoint, focusing solely on self-efficacy risks underestimating the influence of external constraints on learning autonomy. The present findings contribute to this debate by suggesting that self-efficacy operates as an enabling mechanism, influencing how students engage with available learning opportunities rather than determining learning outcomes independently. This perspective integrates psychological and structural dimensions, offering a more balanced understanding of SDLR.

A central contribution of this study is the identification of self-efficacy as a partial mediator in the relationship between perceived academic mentorship and SDLR. This finding indicates that the association between mentorship and SDLR is not solely direct, but is also transmitted through students’ beliefs in their capacity to manage learning demands. In this sense, mentorship appears to function not only as a source of academic guidance, but also as a mechanism through which students develop confidence in their ability to engage in autonomous learning. This interpretation is supported by evidence suggesting that mentorship experiences—particularly those involving feedback, role modeling, and encouragement—can enhance self-efficacy by reinforcing perceptions of competence and control over learning tasks [37, 38].

At the same time, the mediation effect was partial rather than complete, indicating that self-efficacy does not fully account for the relationship between mentorship and SDLR. This suggests the presence of additional pathways through which mentorship may influence learning readiness. For example, prior research has highlighted the role of mentorship in fostering learning engagement, reflective practice, and professional identity development, all of which may contribute to self-directed learning [25, 40]. Conversely, some studies propose that mentorship effects may operate primarily through behavioral modeling and structured guidance rather than internal psychological mechanisms [45]. The present findings help reconcile these perspectives by suggesting that mentorship likely operates through multiple, interacting pathways, including both psychological and behavioral processes.

Overall, the findings suggest that the relationship between academic mentorship and self-directed learning readiness is best understood as a multidimensional process, in which students’ perceptions of mentorship interact with psychological factors such as self-efficacy to influence learning behavior. By demonstrating both direct and indirect associations within a single framework, this study extends existing literature and provides a more comprehensive understanding of how academic mentorship may be linked to the development of self-directed learning readiness in nursing education.

### Implications of the study

The findings of this study offer several implications for nursing education, mentorship practices, and institutional planning, particularly in relation to students’ readiness for self-directed learning. The observed direct and indirect associations between perceived academic mentorship, self-efficacy, and self-directed learning readiness suggest that readiness for autonomous learning may be shaped through an interaction of relational and psychological factors, rather than representing a fixed individual attribute.

From an educational perspective, the findings indicate that academic mentorship may function as more than a supportive activity, and can be understood as part of the broader learning environment through which students engage with and develop learning-related capabilities. In this context, mentorship may contribute to students’ readiness for self-directed learning when it is experienced as supportive, interactive, and conducive to gradual development of independence.

These findings may be interpreted within a conceptual perspective of autonomy-supportive academic mentorship, in which mentorship extends beyond directive guidance to support students’ development of confidence and independent learning capabilities. Within this perspective, mentorship interactions that involve constructive feedback, encouragement, and opportunities for reflection may be associated with stronger self-efficacy, which in turn relates to students’ engagement in self-directed learning processes. This conceptualization does not imply a fixed or prescriptive model, but highlights key relational and psychological elements that may be relevant when considering mentorship practices in nursing education.

The identified mediating role of self-efficacy further suggests that the relationship between mentorship and learning readiness may operate, in part, through students’ beliefs in their ability to manage learning demands. This highlights the potential relevance of approaches that consider both the relational aspects of mentorship and the psychological experiences of learners. However, given the correlational nature of the study, these interpretations should be considered as indicative rather than causal.

At the institutional level, the findings suggest that differences in students’ perceptions of mentorship may be relevant when examining variation in learning readiness. This may have implications for how mentorship roles are supported and implemented within educational settings, particularly in relation to consistency of mentor–mentee interactions and the learning environment provided. Further research is needed to determine how specific mentorship practices or structures contribute to these outcomes.

### Limitations and future directions

Several limitations should be considered when interpreting the findings of this study. First, the use of a cross-sectional design limits causal inference; although significant associations and mediation effects were identified, the temporal direction among mentorship, self-efficacy, and self-directed learning readiness cannot be definitively established. Longitudinal or experimental studies are needed to confirm causal pathways and examine how these relationships evolve over time. Second, data were collected using self-reported measures, which may be subject to response bias or social desirability effects. Although validated instruments with strong psychometric properties were used, future studies could incorporate objective indicators, observational data, or mixed-methods approaches to strengthen inference.

Third, the study was conducted at a single faculty of nursing, which may limit the generalizability of the findings to other institutional, cultural, or educational contexts. Replication across multiple universities and diverse educational settings is warranted. Additionally, mentorship quality was assessed from the students’ perspective only; future research may benefit from incorporating mentors’ perspectives or dyadic assessments to provide a more comprehensive understanding of mentoring relationships. Although all participants had access to and were proficient in using smartphones, the use of an online survey may still introduce a degree of self-selection bias.

Fourth, the study did not examine the duration, frequency, or specific characteristics of mentor–mentee interactions, such as the extent of scaffolding, feedback intensity, or practical guidance provided. These factors may influence the strength and nature of the relationships observed, particularly in relation to self-efficacy development, and should be explored in future research.

Future research should build on the present findings by exploring additional mediating or moderating variables, such as learning engagement, professional identity, psychological capital, or innovation mindset, which may further explain how mentorship influences self-directed learning readiness. Intervention-based studies evaluating structured mentorship programs designed to enhance self-efficacy would also be valuable. Finally, longitudinal follow-up into clinical practice could determine whether gains in self-directed learning readiness during undergraduate education translate into sustained professional learning, competence, and adaptability after graduation.

## Conclusion

This study highlights that self-directed learning readiness among nursing students is a developmental outcome shaped by both mentorship quality and self-efficacy. Effective mentorship directly enhances learning readiness and indirectly strengthens it by fostering students’ confidence in their ability to manage learning demands. Self-efficacy emerges as a critical psychological mechanism linking supportive mentoring relationships to autonomous learning behaviors. These findings emphasize that mentorship initiatives must go beyond guidance to intentionally empower learners, enabling nursing students to become confident, self-directed professionals prepared for continuous learning in complex healthcare environments.

## Data Availability

The raw data supporting the conclusions of this study will be made available by the authors upon reasonable request.
